# Comparative Proteomics of Seminal Exosomes Reveals Size-Exclusion Chromatography Outperforms Ultracentrifugation

**DOI:** 10.3390/biomedicines13102459

**Published:** 2025-10-09

**Authors:** Ajaya K. Moharana, Manesh Kumar Panner Selvam, Soumya Ranjan Jena, Partha K. Chandra, David W. Busija, Luna Samanta, Suresh C. Sikka

**Affiliations:** 1 Department of Urology, Tulane University School of Medicine, New Orleans, LA 70112, USA; 2 Redox Biology & Proteomics Laboratory, Department of Zoology, Ravenshaw University, Cuttack 753003, India; 3 Department of Pharmacology, Tulane University School of Medicine, New Orleans, LA 70112, USA; pchandr1@tulane.edu (P.K.C.);; 4 Tulane Brain Institute, Tulane University, New Orleans, LA 70118, USA

**Keywords:** seminal plasma, extracellular vesicles, exosomes, size-exclusion chromatography (SEC), proteomic profiling

## Abstract

**Background**: Extracellular vesicles, particularly exosomes, play a crucial role in cell–cell communication and as carriers of biomarkers. However, their use in clinical settings is limited due to a lack of standardized isolation and characterization. Ultracentrifugation (UC) is considered a gold standard for exosome isolation but presents several limitations. Size-exclusion chromatography (SEC) has recently gained attention as a superior method, which offers better yield, purity, and protection of exosome physical properties. This study focused on optimizing the SEC method for isolation of exosomes from seminal plasma and comparing yield, quality, and proteome profiles with those obtained by UC. **Methods**: In this SEC method, seminal plasma (0.5 mL) was loaded onto a SEC column and collected in 13 fractions of 0.4 mL each. The physical and molecular characterization of exosomes was carried out using a ZetaView analyzer and Western blot, respectively. Further, SEC-isolated exosomes were used for proteomic profiling and functional bioinformatic analysis. **Results**: The second and third fractions had the highest concentration of exosomes with uniform size and strong expression of exosome markers. Also, comparative proteomic analysis identified 3315 proteins in SEC-isolated exosomes and 931 in UC-isolated exosomes, with 709 proteins in common. SEC-isolated exosomes showed greater overlap with Vesiclepedia’s and ExoCarta’s top 100 lists than UC-isolated exosomes (Vesiclepedia: 91 vs. 77 proteins, ExoCarta: 94 vs. 79). Proteins from SEC- and UC-isolated exosomes showed similar enrichment profiles across all three gene ontology categories. **Conclusions**: Overall, this optimized SEC protocol is a reliable alternative method to isolate seminal exosomes with high purity, supporting its potential applications in clinical and basic research.

## 1. Introduction

Extracellular vesicles (EVs)—apoptotic bodies, microvesicles, and exosomes—play crucial roles in both healthy and disease states [1,2]. These EVs are categorized based on size and origin [3], with exosomes (50–200 nm) derived from the endocytic pathway, while microvesicles (0.1–1 µm) and apoptotic bodies (1–5 µm) are formed by membranous extrusions [4]. In addition, EVs are produced and released through specific mechanisms, allowing them to carry diverse molecular cargo and exert biological effects [2]. These mechanisms have been shown to influence immunological regulation [5], tumor progression [6,7], neurological disorders [8], viral infections [9], and other infectious diseases [10]. In human semen, exosome-like vesicles, known as prostasomes (30–500 nm size), thought to be derived from the prostate gland [11,12,13], are currently called seminal EVs, including exosomes and microvesicles derived from various male reproductive organs [12,14,15,16].

The transfer of seminal EV contents such as miRNA, proteins, metabolites, and lipids to spermatozoa leads to alterations in the biological characteristics and sperm membrane composition [17], thereby impacting sperm biology and function [18]. These modifications encompass sperm maturation [16], enhance sperm motility [19], influence capacitation and the acrosome reaction [20,21], and affect fertilization [22]. These seminal EVs also offer protection to spermatozoa against acidic, inflammatory, and oxidative environments [23]; stabilize the sperm plasma membrane [24]; and exhibit potential anti-human immunodeficiency virus activity [25]. Thus, these seminal exosomes play an important role in sperm vitality and function. In order to evaluate their functions and related mechanisms, it is very important to isolate good-quality pure EVs or exosomes from seminal plasma of normal and infertile men.

Differential ultracentrifugation (UC), size-exclusion chromatography (SEC), ultrafiltration, precipitation, immunoaffinity capture, and microfluidics [3] are used to isolate exosomes and other EVs. UC is considered the research standard and separates particles by density, size, and centrifugal force [1,3]. However, UC is a time-consuming process for exosome isolation and requires expensive equipment maintenance and may damage exosomes [3,25]. Each isolation method comes with its own strengths and weaknesses. Each method yields an isolated product that differs in resolution with respect to vesicle subtype (e.g., exosomes vs. microvesicles), including protein aggregate and high-density lipoprotein contaminations (HDL) [3,26]. SEC has emerged as a more efficient method, which uses a porous gel filtration polymer that allows larger particles to elute first, followed by smaller vesicles and non-exosomal proteins [27,28]. This technique minimizes contamination of plasma proteins and provides high-purity exosomes with an average processing time of 20 min [29,30]. SEC columns have been successfully used to isolate EVs and exosomes from a variety of biological fluids, including plasma, serum, urine, saliva, tears, cerebrospinal fluid, and seminal fluid [31,32,33].

The Izon qEV original SEC columns are effectively used to separate EV particles larger than the matrix pore size from other impurities like high-density lipoproteins [34]. The qEV column has also been used to isolate exosomes from various body fluids of female reproductive organs, such as human vaginal fluid [35] and ovarian follicular fluid [36]. However, there is little information on its application to human seminal fluid and subsequent usefulness for application to sperm biology and function. In this study, we describe seminal exosome isolation based on SEC using the qEV column compared with those isolated by ultracentrifugation, the standard exosome isolation research technique.

## 2. Materials and Methods

### 2.1. Study Participants and Semen Analysis

Semen samples from healthy donors (*n* = 8) were used to standardize SEC qEV column-based seminal exosome isolation. For comparative analysis between SEC and UC isolation techniques, semen samples from a separate cohort of healthy donors (*n* = 3) were employed. Following 2–3 days of sexual abstinence, semen samples were collected and allowed to liquefy at 37 °C for 20–30 min. Semen volume, sperm concentration, motility, and morphology were assessed according to the World Health Organization (WHO) guidelines [37,38]. After semen analyses, a differential centrifugation protocol was employed. Samples were first centrifuged at 1600× *g* (7 min, 37 °C) to separate seminal plasma from sperm, followed by high-speed centrifugation of the plasma fraction at 12,000× *g* (30 min, 4 °C) to ensure complete removal of cellular contaminants. The clear seminal plasma was transferred into new cryovials and stored at −80 °C.

### 2.2. Isolation of Seminal Exosomes Using qEV Columns Coupled with Automatic Fraction Collector (AFC)

**Figure 1:**
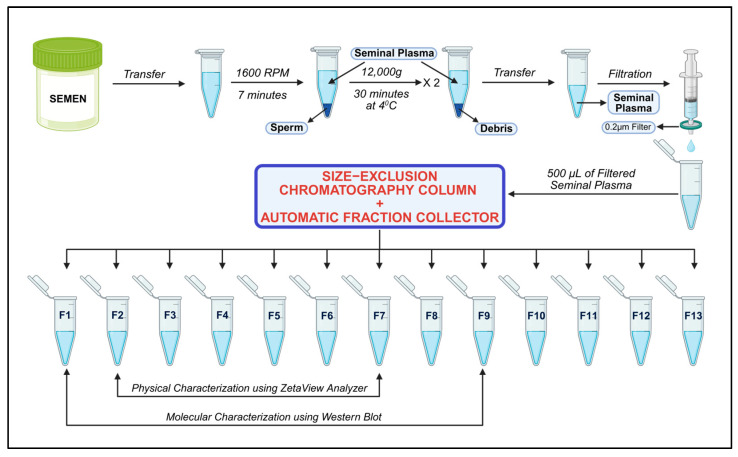
Schematic overview of the SEC-based isolation procedure for purifying exosomes from human seminal plasma. Figure generated with BioRender.com.

Seminal plasma was thawed at 37 °C for 20 min and then subjected to high-speed centrifugation at 12,000× *g* (30 min at 4 °C) to remove residual debris. The clear supernatant was then sequentially filtered through 0.45 µm and 0.22 µm filters. A qEV Gen 2–35 nm column (Izon Science, Christchurch, New Zealand) was adjusted to an AFC preset to collect 13 fractions (0.4 mL each) over a 2.5 mL buffer volume. After equilibrating the qEV 35 nm column with 17 mL [37,38] of phosphate-buffered saline (PBS), 0.5 mL of filtered seminal plasma was loaded into the column. When the sample reached the upper frit, 8 mL of freshly prepared, 0.22-µm-filtered PBS was added. Following buffer collection, 13 fractions of 0.4 mL each were collected. After fractionation, the column was cleaned with 8.5 mL of 0.5 M NaOH, rinsed with 17 mL of filtered PBS, and stored for future use. Figure 1 shows the schematic representation of seminal exosome isolation by SEC qEV columns.

### 2.3. Molecular Characterization of SEC-Isolated Exosomes Using Western Blot

Briefly, 40 µL of Invitrogen™ total exosome isolation reagent (Catalog#4484450, Thermo Fisher Scientific, Waltham, MA, USA) was added to 200 µL of SEC-isolated fractions F1–F9 and incubated at room temperature (20–25 °C) for 10 min. Samples were then centrifuged at 10,000× *g* for 5 min at room temperature. The exosome pellets were resuspended in 25 µL of radio-immunoprecipitation assay (RIPA) buffer (Sigma-Aldrich, St. Louis, MO, USA) containing protease inhibitor cocktail (cOmplete™ ULTRA Tablets, EDTA-free, Roche, Mannheim, Germany) at 4 °C for 1 h to lyse the exosomes. Following centrifugation of the lysates at 12,000× *g* for 30 min at 4 °C, the supernatant was collected and transferred to a fresh tube. Protein concentrations were determined using the Pierce BCA Protein Assay kit (Thermo Fisher Scientific, Waltham, MA, USA). Subsequently, 10 µg of protein from each sample was resolved by SDS-PAGE electrophoresis on 4–20% gradient Mini-PROTEAN TGX Precast Gels (Bio-Rad, Hercules, CA, USA) and transferred to a membrane using the Trans-Blot^®^ Turbo™ Transfer System (Bio-Rad). Membranes were blocked with 5% nonfat milk in Tris-buffered saline-Tween 20 (TBS-T) for at least 1 h at room temperature then incubated with primary antibodies overnight at 4 °C. Primary antibodies: ALIX mouse mAb (Catalog #3A9, Cell Signaling Technology, Danvers, MA, USA) and CD63 Rabbit pAb (Catalog #A5271, ABclonal Technology, Woburn, MA, USA) were used at 1:5000 and 1:1000 dilution, respectively. Secondary antibodies: HRP Goat Anti-Mouse IgG Ab (Catalog #AS003, ABclonal Technology, Woburn, MA, USA) and HRP-conjugated Goat anti-Rabbit IgG (Catalog #AS014, ABclonal Technology, Woburn, MA, USA) were used at 1:10,000 and 1:10,000 dilution, respectively. Polyvinylidene difluoride (PVDF) membranes were subjected to total protein staining for data normalization.

### 2.4. Characterization of SEC Isolated Exosomes by the ZetaView Particle Metrix System

Exosome size, concentration, and zeta potential were determined using a ZetaView PMX-430-Z QUATT laser system (Particle Metrix, Meerbusch, Germany) operating at wavelengths of 405/488/520/640 nm. The system featured a fixed cell assembly and was controlled by ZetaView v8.05.16 SP3 software, as described previously [39]. System calibration and alignment were performed using 100 nm polystyrene standard particles diluted (1:250,000) in aqueous suspension (Applied Microspheres, Leusden, The Netherlands). Prior to analysis, samples were equilibrated to room temperature for 30–45 min. Subsequently, samples were diluted to 1:1000 in deionized distilled water to obtain particle concentrations appropriate for ZetaView Particle Metrix analysis. All measurements were conducted under the same conditions (room temperature, pH 7.0, sensitivity 80, shutter speed 100), with measurements taken at 11 positions for each replicate. Each sample was measured in triplicate, and outlier positions were automatically excluded.

### 2.5. Isolation of Seminal Exosomes Using UC

The seminal plasma (*n* = 3), devoid of sperm, was centrifuged at 1600× *g* for 7 min, followed by 12,000× *g* for 30 min at 4 °C to remove residual spermatozoa and debris. The seminal plasma containing seminal exosomes was sequentially filtered through 0.45 µm and 0.22 µm filters. The filtrate (0.5 mL) was diluted with 2.0 mL 1× PBS, layered over a 2 mL cushion of 30% sucrose in 20 mM Tris buffer (pH 7.4), and subjected to UC at 100,000× *g* for 90 min at 4 °C using an SW 28 swinging-bucket rotor (Beckman Coulter, Brea, CA, USA), as described previously [40]. The pellets were washed three times with PBS, resuspended in 0.2 mL PBS, and stored at −80 °C until further use.

Seminal plasma (*n* = 3) from the same donors was used to isolate exosomes using qEV columns, as mentioned in Section 2.2. The SEC-isolated exosome fractions F2 to F7 were pooled and used for further comparative analysis with the UC-isolated exosome fraction.

### 2.6. Exosome Characterization by Scanning Electron Microscopy (SEM)

Scanning electron microscopy (SEM) was used to confirm the seminal exosome morphology isolated using both SEC and UC methods. Exosome samples were diluted with PBS to the appropriate concentration, and approximately 25–40 µL were fixed onto coverslips with 4% formaldehyde for 15 min at room temperature to preserve their morphology for subsequent preparation steps. To remove water content, coverslips were placed in an incubator overnight at 60 °C for evaporation. SEM requires an electrically conductive sample for optimal imaging, and both exosomes and coverslip substrates have low electron density; this can lead to poor image quality, sample charging, damage, and carbon deposition. Therefore, gold in 1 nm layers (10 cycles of 10 mA for 30 s each) for a resulting 10 nm gold coating was applied using the JFC-1300 Auto Fine Coater (JEOL, Tokyo, Japan) [41].

### 2.7. Protein Extraction, Quantification, and Western Blot

Exosomes isolated using UC and SEC methods were lysed in RIPA buffer supplemented with Protease Inhibitor Cocktail (cOmplete™ ULTRA Tablets, EDTA-free, Roche, Mannheim, Germany) [41]. Lysates were clarified by centrifugation, and protein concentrations were determined by the BCA assay (BCA: Thermo Fisher Scientific, Waltham, MA, USA).

For Western blot analysis, 10 µg of protein from exosomes isolated by SEC and UC methods were electrophoresed on SDS-PAGE. The proteins from the gel were then transferred onto a PVDF membrane using the Trans-Blot Turbo system, as described earlier in Section 2.3. The membrane was probed with rabbit anti-TSG101 antibody (Catalog #Ab275018, ABclonal Technology, Woburn, MA, USA) at a 1:1500 dilution, followed by incubation with HRP-conjugated goat anti-rabbit IgG (Catalog #11-315, Abgenex, Fremont, CA, USA) at a 1:1500 dilution.

### 2.8. Proteome Profiling of SEC-Isolated Exosomes

For proteomic analysis, SEC-isolated seminal exosome samples (*n* = 3), were prepared in RIPA lysis buffer. Samples were centrifuged at 16,000× *g* for 10 min at 4 °C, and supernatants were collected. Aliquots of 100 μg from each sample were reduced with Tris(2-carboxyethyl)phosphine (TCEP), alkylated with iodoacetamide, and precipitated with acetone overnight at −20 °C. Pellets were centrifuged at 8000× *g*, air-dried, and resuspended in triethylammonium bicarbonate buffer (TEAB). Each sample was digested with 2 μg trypsin (Promega) at 37 °C for 16 h. Peptides were desalted using styrene divinylbenzene reverse phase sulfonate (SDB-RPS) StageTips, and 20 μg from all samples were pooled for strong cation exchange (SCX) fractionation. SCX was performed with stepwise elution using increasing concentrations of ammonium acetate (50–300 mM in 20% acetonitrile [ACN], 0.2% formic acid [FA]) for the first five fractions and 5% ammonium hydroxide in 80% ACN for the final step. Samples and fractions were vacuum-dried and reconstituted in 8 μL of 2% ACN and 0.1% FA for liquid chromatography–mass spectrometry (LC–MS) analysis.

The analysis was conducted using an Ultimate 3000 nanoLC system coupled by a nano- electrospray ion source to a Q Exactive HFX Orbitrap (Thermo Fisher Scientific, Bremen, Germany). Spectral library construction involved loading SCX fractions onto a C18 trap column (100 μm I.D. × 2.5 cm) followed by separation on a PepMap RSLC C18 analytical column (2 μm, 75 μm × 25 cm) with solvent A (0.1% FA in water). Peptides were eluted with a linear gradient (5–30% buffer B) over 80 min, ramped to 60% over 4 min, then to 95% over 8 min, followed by re-equilibration to 5% (total runtime: 106 min).

Data were acquired using data-dependent acquisition (DDA) and data-independent acquisition (DIA) methods and processed with Spectronaut and Perseus platforms (Version 18). Initially, all DDA datasets were processed and consolidated into a comprehensive spectral library using Spectronaut software for the identification of specific tryptic peptides present in the reference proteomes. The resulting spectral library was then utilized by Spectronaut for subsequent DIA data analysis. Finally, the Perseus platforms were used for the subsequent downstream analysis.

### 2.9. Comparative Proteomic Analysis

The proteome profile of SEC-isolated exosomes was compared with the proteins detected in the UC-isolated exosome [40]. Furthermore, we validated the proteomes of both SEC- and UC-isolated exosomes with public extracellular vesicle databases such as Vesiclepedia and ExoCarta [42,43]. In addition, functional enrichment analysis of seminal exosomal proteins was performed using the Database for Annotation, Visualization, and Integrated Discovery (DAVID) (v2024q2).

### 2.10. Statistical Analysis

All the variables were tested for normal distribution, and either parametric (paired-samples *t*-test) or non-parametric (Wilcoxon test) tests were employed. The data were shown as mean ± standard deviation (SD). Statistical analysis was performed using MedCalc statistical software version 23.1.7 (MedCalcSoftware bv, Ostend, Belgium), and *p* < 0.05 was considered statistically significant.

## 3. Results

### 3.1. Detection of Exosome-Specific Markers in Fractions Isolated Using qEV Columns

**Figure 2:**
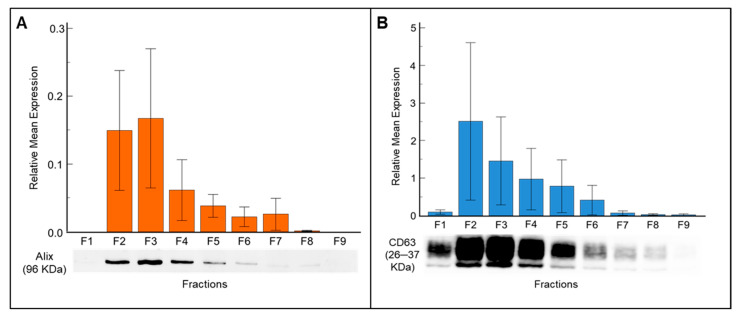
Western blot analysis demonstrates the expression of exosome-specific markers in seminal plasma-derived exosome fractions isolated by SEC. (**A**) Detection of ALIX (96 kDa), an endosome marker, across fractions F1–F9. (**B**) Expression profile of CD63 (26 kDa), an exosome surface marker, in the same fractions. Data are presented as mean ± SD.

Fractions F1 to F9 of each sample were analyzed to assess the presence and expression levels of exosome-specific markers such as ALIX and CD63. ALIX was consistently detected in fractions F2 to F7 (Figure 2A), whereas CD63 was expressed across all fractions (Figure 2B). The presence of high intensity bands for ALIX protein in fractions F2 and F3 indicates that these two fractions were enriched with exosomes. The relative mean expression of ALIX compared with total protein in fractions F2 and F3 was 0.14 ± 0.25 and 0.16 ± 0.29, respectively (Figure 2A).

The relative mean expression of CD63 corresponded with fraction number, with the highest expression observed in fraction F2, followed by fractions F3, F4, and F5 (Figure 2B). From fraction F6 onward, although the total protein amount increased, the band intensity decreased, suggesting that non-exosome proteins became more abundant, thus reducing the purity of exosomes. Fraction F1 showed no ALIX expression, and the CD63 band intensity was faint as expected, since this fraction contains the flow through (void volume) buffer. Further, the exosome specific marker CD81 exhibits expression patterns similar to those of ALIX and CD63 (Supplementary Figure S1).

### 3.2. Physical Characteristics of Seminal Exosomes Isolated Using qEV Columns

**Figure 3:**
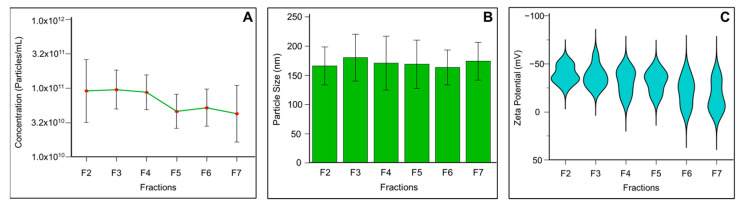
Physical and electrokinetic characterization of seminal plasma-derived exosomes isolated using SEC and analyzed by the ZetaView Particle Metrix nanoparticle tracking analysis system. (**A**) Particle concentration measurements displaying the number of exosome particles per milliliter across purified fractions. (**B**) Size distribution analysis showing the diameter profile of exosomes. (**C**) Zeta potential analysis revealing the surface charge characteristics of exosomes. Data are presented as mean ± SD.

Based on the detection of exosome specific markers, fractions F2 to F7 were considered for particle analysis using a ZetaView Particle Metrix System to assess particle concentration, charge, and size range. Particle number was negligible in fraction 1 across all samples, corresponding to the column void volume. Thus, fraction F1 was excluded from particle analysis. The mean concentration of exosomes per mL in fractions F2 to F7 is presented in Figure 3A. Fractions F2, F3, and F4 showed the highest concentrations of exosomes, with 1.5 ± 1.7 × 10^11^, 1.14 ± 0.6 × 10^11^, and 1.02 ± 0.63 × 10^11^ particles/mL, respectively. In contrast, exosome concentrations in fractions F5 to F7 were relatively low. Moreover, fractions F8 to F13 were found to contain protein contaminants, confirmed by Western blot, showing fewer exosomes in these fractions (data not presented). Consequently, higher fractions were excluded from zeta analysis.

The exosome size distribution revealed a median size across fractions F2 to F7 ranging from 151.8 to 166.4 nm. Figure 3B shows a relatively homogeneous exosome population in human seminal fluid in the isolated vesicles. The zeta potential values indicate that mean particle charge increased with fraction number, ranging from −40 to −15 mV across fractions F2 to F7 (Figure 3C).

### 3.3. Comparative Analysis: Physical, Morphological, and Molecular Characteristics of Seminal Exosomes Isolated Using SEC and UC Methods

The concentration, size, and zeta potential of seminal exosomes were compared between samples isolated using UC and SEC methods. In terms of exosome concentration (Table 1), the SEC method yielded a significantly higher number of particles (1.1 ± 0.12 × 10^11^ particles/mL) compared to the UC technique (1.8 ± 0.03 × 10^8^ particles/mL). Similarly, the mean diameter of exosomes isolated using SEC was 148.3 ± 0.69 nm, whereas those isolated by UC had a mean diameter of 174.4 ± 17.16 nm (Table 1). This indicates that exosomes isolated by the SEC method yielded a greater number of smaller-sized vesicles compared to the UC method, although the average values did not differ significantly (*p* > 0.05). Exosomes isolated by either protocol showed comparable zeta potential values (Table 1).

**Figure 4:**
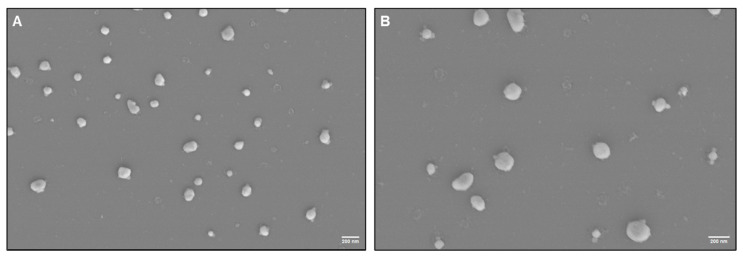
Ultrastructural characterization of seminal plasma-derived exosomes by scanning electron microscopy (SEM) isolated using the (**A**) SEC and (**B**) UC methods. Scale bar, 200 nm.

SEM was used to characterize the seminal exosome morphology isolated using SEC and UC methods. SEM images of seminal exosomes obtained using SEC and UC methods revealed typical morphology of EVs, with diameters ≤ 200 nm (Supplementary Table S1). The samples appeared uniform, with minimal debris contamination (Figure 4). Expression of the exosome-specific markers TSG-101 and CD81 was detected in exosomes isolated using both UC and SEC methods (Supplementary Figure S2).

### 3.4. Comparative Proteome Profile of SEC Versus UC Isolated Exosomes

**Figure 5:**
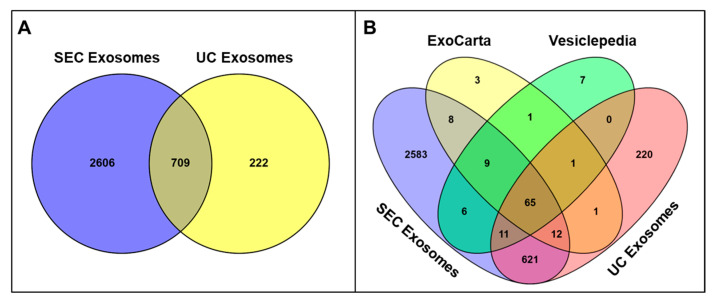
(**A**) Quantitative comparison showing the total number of proteins identified in exosome samples purified by SEC versus UC isolation protocols, demonstrating the protein recovery efficiency of each purification method. (**B**) Venn diagram analysis comparing the protein profiles of SEC- and UC-isolated seminal exosomes with established extracellular vesicle protein databases (Vesiclepedia and ExoCarta) to assess the overlap with known exosome-associated proteins. Venny 2.0 was used to generate Venn diagrams.

A total of 3315 proteins were identified in SEC-isolated seminal exosomes, whereas only 931 proteins were reported in exosomes isolated using the UC technique, with 709 proteins common to exosomes isolated with both techniques. We also noticed that 2606 and 222 unique proteins were included in exosomes isolated by SEC and UC methods, respectively (Figure 5A and Supplementary Table S2). Bioinformatic analysis showed that 91 proteins detected in SEC-isolated exosomes and 77 found in UC-isolated exosomes matched proteins from Vesiclepedia’s top 100 list (Figure 5B and Supplementary Table S3). Similarly, 94 of the top 100 proteins listed in Exocarta were identified in SEC-isolated exosomes, while 79 were found in UC-isolated exosomes (Figure 5B and Table S3). Particularly, exosome markers such as CD81, CD63, and PDCD6IP or ALIX were ranked differently based on the number of identified peptides between SEC and UC fractions, whereas proteins such as GAPDH and ANXA2 had similar rankings across both methods (Supplementary Table S4).

**Figure 6:**
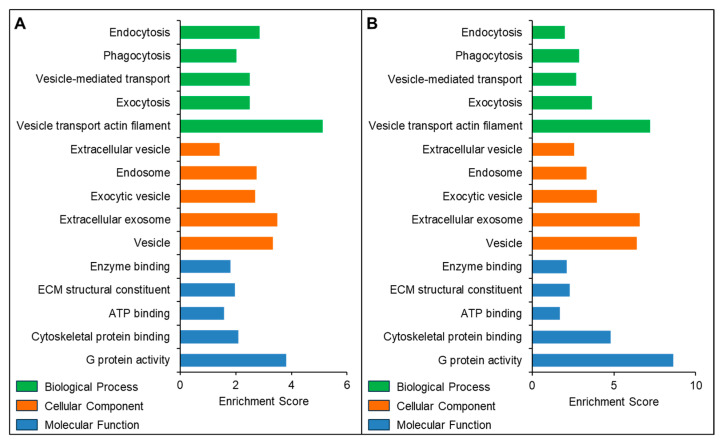
Gene Ontology and functional enrichment analysis of proteins identified in seminal plasma-derived exosomes isolated using the (**A**) SEC and (**B**) UC methods.

The Gene Ontology (GO) enrichment results revealed that the proteins identified in both SEC- and UC-isolated exosomes were enriched in similar cellular components (CC), biological processes (BP), and molecular functions (MF). The top 5 GO terms for CC, MP, and BP-associated proteins from both SEC- and UC-isolated exosomes are presented in Figure 6.

## 4. Discussion

A semen sample is an ideal “liquid biopsy” for assessing biomarkers of male reproductive health [44]. Semen EVs play important roles in sperm motility, capacitation, and the acrosome reaction—the essential steps for fertilization—though their precise contributions remain unclear [22,45,46]. Interest in EVs, especially exosomes as biomarkers, has spurred advancements in isolation methods [47]. Exosome isolation is commonly carried out via UC, but this multistep method is labor-intensive, requires an expensive ultra-high-speed centrifuge requiring regular maintenance, and may damage vesicles [48,49]. Alternative methods, like magnetic bead precipitation, do not require expensive, labor-intensive, or spacious equipment, but they often result in heterogeneous exosome populations with contaminants [50,51]. Thus, refining exosome isolation methods is key to ensuring vesicle purity and vesicle profile maintenance to evaluate their proper role. SEC offers an alternative approach that preserves exosome integrity without requiring centrifugal force, thus yielding higher-quality exosomes compared with traditional methods. Furthermore, SEC has proven particularly effective for serum and plasma exosome isolation [50,52]. In this study, we effectively optimized SEC to isolate exosomes from human seminal plasma. SEM confirmed the presence of vesicles with less than 200 nm diameter in SEC fractions. Our Western blot results also indicated that the exosome markers ALIX and CD63 were highly expressed in fractions F2 and F3, confirming an abundance of exosome-like vesicles. Expression of CD63 from fractions F2 through F6 indicates a predominance of small EVs. Focusing on individual SEC fractions, we identified enriched exosome fractions suitable for pooling to improve yield without compromising purity. Additionally, SEC demonstrates superior capability in separating exosomes from protein aggregates.

Quantifying exosomes derived from seminal fluid presents significant challenges due to the inherent variability in semen composition obtained from different individuals [53]. The SEC technique provides gentler processing conditions that preserve exosome membrane integrity [54]. In this current study, ZetaView Particle Metrix technology revealed that fractions 2 to 4 from the SEC procedure contained most of the isolated seminal exosomes, indicating that SEC effectively concentrated a homogeneous population of seminal exosomes. Understanding the dimensional properties of seminal exosomes provides crucial insights into male reproductive health and fertility assessment. The size of the seminal exosomes affects their cellular uptake, tissue distribution, and biological activity [55], while simultaneously providing valuable diagnostic information about male reproductive health. The seminal exosomes may display unique size heterogeneity due to their diverse cellular origins within the male reproductive tract [56]. Our optimized SEC protocol isolated exosomes with a mean diameter of 148.3 nm, which aligns closely with exosomes derived from other biological fluids using the same technique. In conjunction with size parameters, zeta potential constitutes a fundamental indicator of exosome functional integrity and biological efficacy. In general, nanoparticles with zeta potential between −20 and +20 mV tend to aggregate [57]. Zeta potential of seminal exosomes isolated using SEC was determined as −32.91 mV, suggesting the presence of stable seminal exosomes with minimal aggregation risk [58]. For functional studies requiring biologically active seminal exosomes, SEC provides superior techniques to protect membrane integrity.

The proteomic profile of seminal exosomes offers insights into sperm function, intercellular communication, and disease biomarkers [59]. Exosomal integrity is a prerequisite for accurate proteomic analysis. Damaged or lysed vesicles compromise data quality, biological interpretation, and reproducibility. In our study, the proteome profile of SEC-isolated seminal exosomes revealed the presence of specific protein markers such as tetraspanins (CD63, CD81, CD9) and Rab GTPases, suggesting endosomal origin. Most proteins from the Vesiclepedia (91 out of the top 100) and ExoCarta (94 out of the top 100) databases were also detected in our SEC-isolated exosomes. The 91% overlap with Vesiclepedia and 94% overlap with ExoCarta suggest that SEC may provide better enrichment of canonical exosomal proteins, potentially due to its gentler separation mechanism that preserves intact vesicles and reduces co-purification of non-exosomal protein complexes, unlike those separated by the UC technique. Since UC remains the older and most common method for exosome isolation, we compared its efficiency with our optimized latest SEC protocol. Functional enrichment analysis revealed similar biological patterns in SEC and UC isolated exosomes. SEC-isolated exosomal proteins could be associated with different populations of extracellular vesicles and endosomes. In addition, these seminal exosomal proteins were enriched in biological processes such as endocytosis, vesicle-mediated transport, and exocytosis, as identified by our proteomics data. Our findings align with similar GO terms reported in EVs isolated with ultrafiltration SEC and UC methods, although they did not assess whether EV patterns differed from freely secreted proteins [60]. These proteomics results suggest that despite some differences, our optimized SEC protocol was able to isolate and capture biologically relevant exosomes not only suitable for proteomic but also molecular and other functional biomarker research. These comparative findings validate the rising trend toward SEC use in exosome studies.

Despite many strengths, our study had few limitations. We did not confirm the absence of large EVs or specific protein contaminants in our samples. Although SEC effectively isolates exosomes from body fluids with minimal contaminants, the qEV original 35 nm column’s maximum volume capacity of 500 µL restricts scalability for high-throughput applications. However, our protocol, combining 0.22 μm filtration with SEC, yielded exosomes with sizes between 162 and 172 nm. Fractions F2 and F3 had the highest exosome concentration and marker intensity, making them ideal fractions for downstream exosome isolation and functional analysis.

As clinical research increasingly emphasizes biomarker discovery, SEC’s cost-effectiveness and reproducibility highlight its potential role in diagnostic applications. Overall, our study demonstrates that qEV is a novel SEC method that reliably isolates contamination-free exosomes from human seminal plasma, providing high yields of exosomes with significant physiological roles and unique cargo content, thereby rendering them suitable for future research and clinical application for better patient management.

## 5. Conclusions

Various methods have been developed to improve exosome isolation with variable purity and consistency. The findings from our study confirm the effectiveness of the SEC protocol for seminal exosome isolation and characterization. Furthermore, we also present a standardized, efficient method for isolating exosomes from human seminal plasma, yielding a homogenous population in size, concentration, charge, and protein composition. This method is compatible with both qualitative and quantitative downstream analysis, offering a faster and higher-yield alternative to existing techniques like UC, with the potential to advance exosome research.

## Figures and Tables

**Table 1:** Comparative yield and purity of exosomes isolated by SEC and UC methods as determined by the ZetaView Particle Metrix system.

Parameters	SEC Exosomes (*n* = 3)	UC Exosomes (*n* = 3)	*p* Value
Concentration (Particles/mL)	1.1 ± 0.1 × 10^11^	1.8 ± 0.03 × 10^8^	0.0001
Mean diameter (nm)	148.3 ± 0.69	174.4 ± 17.16	0.0576
Zeta potential (mV)	−32.91 ± 5.29	−33.01 ± 1.96	0.9772

Note: Values reported as mean ± SD.

## Data Availability

Data from our study are available from the corresponding author upon reasonable request.

## Supplementary Materials

The following supporting information can be downloaded at: https://www.mdpi.com/article/10.3390/biomedicines13102459/s1, Figure S1: Western blot analysis of CD81 (26 KDa) in seminal exosome fractions (F1 to F9) isolated using SEC method; Figure S2: (A) Western blot analysis of TSG-101 (50 KDa) in exosomes isolated using SEC and UC techniques, (B) Western blot analysis of CD81 (26 KDa) in exosomes isolated using SEC and UC techniques; Table S1: Mean diameter of seminal plasma-derived exosomes particles observed under scanning electron microscope; Table S2: Proteomic profile of seminal exosomes isolated by size-exclusion chromatography and ultracentrifugation techniques; Table S3: Comparative proteomic profile of seminal exosomes isolated by size-exclusion chromatography and ultracentrifugation techniques with public extracellular vesicle databases; Table S4: List of EV marker proteins from “Vesiclepedia” (V) and “Exocarta” (E) databases and their presence or absence in exosomes isolated from seminal plasma using SEC and UC. The number of peptide spectral matches (PSM) gives an estimate of the relative protein abundance.
