# Supplementary material for: Comparative Proteomics of Seminal Exosomes Reveals Size-Exclusion Chromatography Outperforms Ultracentrifugation
Source: Biomedicines. 2025 Oct 9;13(10):2459. doi: 10.3390/biomedicines13102459 (PMC12561774; doi:10.3390/biomedicines13102459)

Supplementary Figure 1: Western blot analysis of CD81 (26 KDa) in seminal exosome fractions (F1 to F9) isolated using SEC method

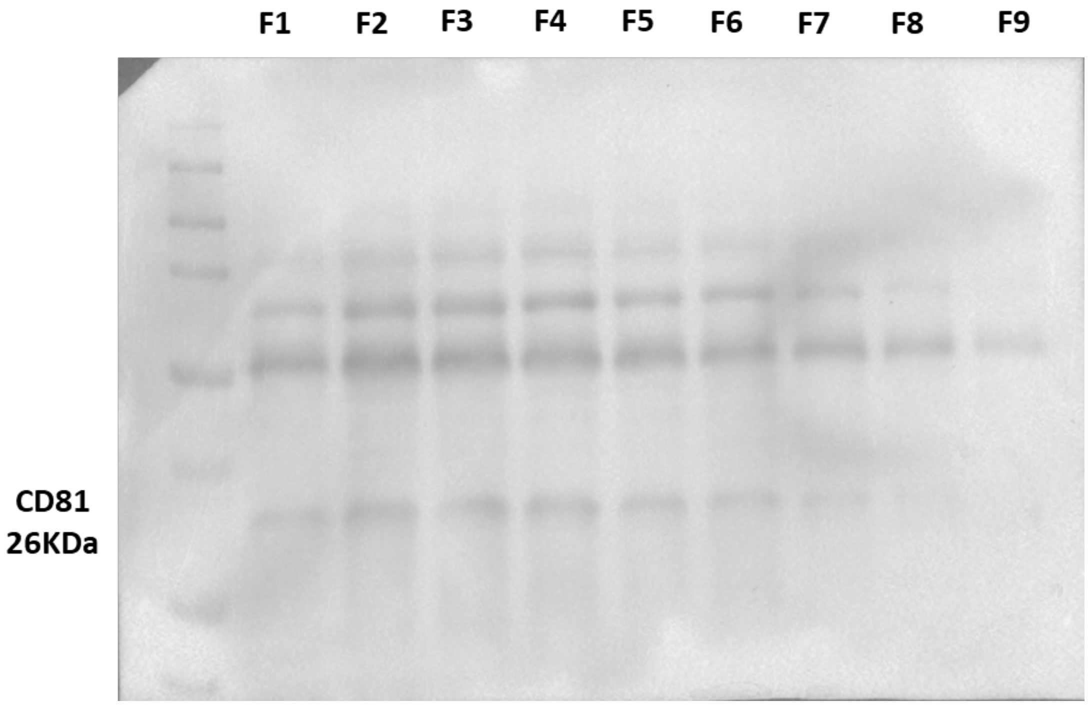

Supplement: Supplementary file 1 [file biomedicines-13-02459-s001.zip › Figure S1.pdf]
