# Supplementary material for: Comparative Proteomics of Seminal Exosomes Reveals Size-Exclusion Chromatography Outperforms Ultracentrifugation
Source: Biomedicines. 2025 Oct 9;13(10):2459. doi: 10.3390/biomedicines13102459 (PMC12561774; doi:10.3390/biomedicines13102459)
Supplement: Supplementary file 1 [file biomedicines-13-02459-s001.zip › Table S1.pdf]

Supplementary Table 1. Mean diameter of seminal plasma-derived exosomes particles observed under scanning electron microscope.

Parameters	SEC Exosomes	UC Exosomes
Mean diameter (nm)	112.5 ± 20.2	130.3 ± 45.5

Note: Values reported as mean ± SD
