# Supplementary material for: Comparative Proteomics of Seminal Exosomes Reveals Size-Exclusion Chromatography Outperforms Ultracentrifugation
Source: Biomedicines. 2025 Oct 9;13(10):2459. doi: 10.3390/biomedicines13102459 (PMC12561774; doi:10.3390/biomedicines13102459)
Supplement: Supplementary file 1 [file biomedicines-13-02459-s001.zip › Table S4.pdf]

Supplementary Table 4: List of EV marker proteins from "Vesiclepedia" (V) and "Exocarta" (E) databases and their presence or absence in exosomes isolated from seminal plasma using SEC and UC. The number of peptide spectral matches (PSM) gives an estimate of the relative protein abundance.

Protein	Associated Databases	SEC-EVs		UC-EVs	
		Present / Absent	# PSM	Present / Absent	# PSM
CD63	V & E	Present	14	Present	5
CD9	V & E	Present	19	Present	1
PDCD6IP	V & E	Present	20	Present	41
TSG101	V & E	Present	18	Present	2
CD81	V & E	Present	15	Present	6
GAPDH	V & E	Present	18	Present	19
FLOT1	V & E	Present	17	Absent	NA
ACTB	V & E	Present	21	Present	34
ANXA2	V & E	Present	19	Present	16
SDCBP	V & E	Present	18	Present	8
HSP90AA1	V & E	Present	19	Present	35
HSPA8	V & E	Present	20	Present	35
ANXA5	V & E	Present	21	Present	19
ENO1	V & E	Present	18	Present	21
PKM	V & E	Present	20	Present	34
HSP90AB1	V & E	Present	20	Present	31
YWHAZ	V & E	Present	19	Present	25
PGK1	V & E	Present	19	Present	21
YWHAE	V & E	Present	18	Present	12
ANXA1	V & E	Present	20	Present	29
VCP	V & E	Present	18	Present	11
PPIA	V & E	Present	19	Present	15
EEF1A1	V & E	Present	18	Present	21
ALB	V & E	Present	18	Present	92
ALDOA	V & E	Present	18	Present	24
ITGB1	V & E	Present	11	Absent	NA
CLTC	V & E	Present	11	Present	6
TPI1	V & E	Present	18	Present	22
CFL1	V & E	Present	17	Present	13
EEF2	V & E	Present	18	Present	32
MSN	V & E	Present	16	Absent	NA
GNB1	V & E	Present	17	Present	5
PRDX1	V & E	Present	20	Present	9
ANXA6	V & E	Present	19	Present	4
SLC3A2	V & E	Present	16	Absent	NA
EZR	V & E	Present	20	Present	31

LDHA	V & E	Present	19	Present	2
LDHB	V & E	Present	18	Present	2
BSG	V & E	Present	15	Absent	NA
CDC42	V & E	Present	19	Present	9
PFN1	V & E	Present	19	Present	9
ATP1A1	V & E	Present	13	Absent	NA
ACTN4	V & E	Present	18	Present	1
HSPA1A	V & E	Present	20	Present	27
YWHAB	V & E	Present	16	Present	11
GNAI2	V & E	Present	16	Present	4
YWHAQ	V & E	Present	17	Present	19
FASN	V & E	Present	20	Present	37
CLIC1	V & E	Present	19	Present	13
PRDX2	V & E	Present	19	Present	11
GSN	V & E	Present	15	Absent	NA
CCT2	V & E	Present	18	Present	9
RAB5C	V & E	Present	17	Present	4
RAP1B	V & E	Present	19	Present	4
GNB2	V & E	Present	19	Present	4
LGALS3BP	V & E	Present	20	Present	20
YWHAG	V & E	Present	17	Present	13
TFRC	V & E	Present	12	Absent	NA
RAN	V & E	Present	17	Present	3
GDI2	V & E	Present	19	Present	14
CCT3	V & E	Present	17	Present	9
AHCY	V & E	Present	18	Present	6
HSPA5	V & E	Present	16	Present	8
ACLY	V & E	Present	17	Present	17
UBA1	V & E	Present	18	Present	19
ANXA11	V & E	Present	20	Present	11
KPNB1	V & E	Present	16	Present	1
RAC1	V & E	Present	14	Present	7
MFGE8	V & E	Present	18	Present	1
TCP1	V & E	Present	17	Present	7
RHOA	V & E	Present	18	Present	9
GNAS	V & E	Present	18	Present	4
CCT5	V & E	Present	16	Present	6
A2M	V & E	Present	13	Absent	NA
FLOT2	V	Present	15	Present	1
MYH9	V	Present	18	Present	21
HIST1H4A	V	Absent	NA	Present	12
RAB10	V	Present	16	Present	6
HLA-A	V	Present	15	Absent	NA
ACTN1	V	Present	15	Present	2
ANXA7	V	Present	18	Present	3

CCT4	V	Present	17	Present	6
C3	V	Present	11	Present	3
TUBB4B	V	Present	20	Present	24
CAP1	V	Present	15	Present	3
TLN1	V	Present	17	Absent	NA
CCT6A	V	Present	16	Present	3
RALA	V	Present	14	Present	2
EHD1	V	Present	17	Present	5
CCT8	V	Present	19	Present	5
PGAM1	V	Present	18	Present	10
IQGAP1	V	Present	17	Absent	NA
VCL	V	Present	15	Present	1
GPI	V	Present	17	Present	4
EIF4A1	V	Present	16	Present	3
RAB7A	V	Present	18	Present	4
EEF1G	V	Present	17	Present	1
ADAM10	V	Present	17	Absent	NA
YWHAZ	E	Present	19	Present	25
TUBA1B	E	Present	20	Present	21
EHD4	E	Present	17	Present	8
RAB7A	E	Present	18	Present	4
ARF1	E	Present	13	Present	2
ACTG1	E	Present	21	Present	34
ANXA4	E	Present	17	Present	11
TUBA1C	E	Present	13	Present	21
RAB14	E	Present	18	Absent	NA
HIST2H4A	E	Absent	NA	Present	12
THBS1	E	Present	13	Absent	NA
RAB5A	E	Present	19	Present	3
PTGFRN	E	Present	12	Absent	NA
RAB5B	E	Present	17	Present	3
RAB1A	E	Present	14	Present	5
LAMP2	E	Present	20	Present	2
ITGA6	E	Present	11	Absent	NA
HIST1H4B	E	Absent	NA	Present	12
TKT	E	Present	15	Present	4
STOM	E	Present	15	Absent	NA
SLC16A1	E	Present	13	Absent	NA
RAB8A	E	Present	15	Present	5
